# Chromosomal organization of repetitive DNAs in *Hordeum bogdanii* and *H. brevisubulatum* (Poaceae)

**DOI:** 10.3897/CompCytogen.v10i4.9666

**Published:** 2016-10-07

**Authors:** Quanwen Dou, Ruijuan Liu, Feng Yu

**Affiliations:** 1 Key Laboratory of Adaptation and Evolution of Plateau Biota, Northwest Plateau Institute of Biology, Chinese Academy of Sciences, Xining 810001, China

**Keywords:** Hordeum bogdanii, Hordeum brevisubulatum, autopolyploid, repetitive sequence, FISH

## Abstract

Molecular karyotypes of *Hordeum bogdanii* Wilensky, 1918 (2n = 14), and Hordeum brevisubulatum Link, 1844 ssp. brevisubulatum (2n = 28), were characterized by physical mapping of several repetitive sequences. A total of 18 repeats, including all possible di- or trinucleotide SSR (simple sequence repeat) motifs and satellite DNAs, such as pAs1, 5S rDNA, 45S rDNA, and pSc119.2, were used as probes for fluorescence in situ hybridization on root-tip metaphase chromosomes. Except for the SSR motifs AG, AT and GC, all the repeats we examined produced detectable hybridization signals on chromosomes of both species. A detailed molecular karyotype of the I genome of *Hordeum bogdanii* is described for the first time, and each repetitive sequence is physically mapped. A high degree of chromosome variation, including aneuploidy and structural changes, was observed in *Hordeum brevisubulatum*. Although the distribution of repeats in the chromosomes of *Hordeum brevisubulatum* is different from that of *Hordeum bogdanii*, similar patterns between the two species imply that the autopolyploid origin of *Hordeum brevisubulatum* is from a *Hordeum* species with an I genome. A comparison of the I genome and the other *Hordeum* genomes, H, Xa and Xu, shows that colocalization of motifs AAC, ACT and CAT and colocalization of motifs AAG and AGG are characteristic of the I genome. In addition, we discuss the evolutionary significance of repeats in the genome during genome differentiation.

## Introduction

Species in Triticeae have large genomes, 75% of which consists of repetitive sequences (Flavell et al. 1974, 1977). Many repetitive sequences, such as microsatellite and satellite DNA, can generate fluorescence in situ hybridization (FISH) patterns on individual chromosomes that are specific to a single species (Tsujimoto et al. 1997, Cuadrado et al. 2008). The FISH patterns generated by these repetitive probes always produce a stable and unique karyotype for a given species (Badaeva et al. 1996, 2002). The FISH patterns from a few repeats correspond to the heterochromatin regions in chromosomes (Cuadrado et al. 1995, Pedersen and Langridge 1997). Thus, the genomic organization of a given species can be characterized using FISH-based cytological analysis. Phylogenies can even be derived from repeat-based comparative FISH karyotyping (Cuadrado and Jouve 2002, Jiang and Gill 2006, Heslop-Harrison and Schwarzacher 2011).

The genus *Hordeum* Linnaeus, 1753 in the tribe Triticeae is divided into 32 species and is distributed in southern South America, South Africa, and the northern hemisphere (Bothmer et al. 1995). Cytotypes in this genus exist at three ploidy levels (diploid, tetraploid and hexaploid) with a basic chromosome number of *x* = 7 (Bothmer et al. 1995). Hybrid analysis and C-band analysis (Bothmer et al. 1986, 1995, Linde-Laursen et al. 1992) have revealed four genome groups in *Hordeum* that are designated as H (*Hordeum bulbosum* Linnaeus, 1756; *Hordeum vulgare* Linnaeus, 1753), Xa (*Hordeum marinum* Hudson, 1778), Xu (*Hordeum murinum* Linnaeus, 1753), and I (all the remaining species) (Blattner 2009). The H genome chromosomes of *Hordeum vulgare* were first characterized using FISH probes of all possible classes of trinucleotide SSRs (simple sequence repeat). This provided detailed information on the sequence content of barley chromatin and saturated the physical map of all the barley chromosomes (Cuadrado and Jouve 2007a). Later, cytogenetic diversity between *Hordeum vulgare* and *Hordeum bulbosum*, was revealed using probes of SSRs, 5S rDNA, 45S rDNA, and 120-bp repeats from *Secale cereale* Linnaeus, 1753 (Carmona et al. 2013a). Karyotypes of the species of the *Hordeum marinum* complex were determined using several tandem repeats. The results revealed the genome structure of different *Hordeum marinum* taxa and demonstrated the allopolyploid origin of tetraploid forms of *Hordeum gussoneanum* Parlatore, 1845 (Carmona et al. 2013b). In addition, the genomic constitution of *Hordeum murinum* was characterized using multiple repetitive sequences. The results identified all the individual chromosomes within the *Hordeum murinum* complex, elucidated its genomic structure and phylogeny, and explained the appearance of different cytotypes (Cuadrado et al. 2013). Thus far, detailed information on the chromosome organization of multiple repetitive sequences is available for the H, Xa, and Xu genomes. However, this information is still lacking for the I genome.

*Hordeum bogdanii* Wilensky, 1918, and *Hordeum brevisubulatum* , should include the I genome based on the description of Blattner (2009). *Hordeum bogdanii* is a rather common Asiatic species with a distribution that ranges from western Iran to eastern China and a diploid form of 2n = 2x = 14 (Yang et al. 1987). Conversely, the *Hordeum brevisubulatum* complex is distributed from Western Turkey to eastern China and consists of diploids, tetraploids, and hexaploids (Bothmer 1979, Linde-Laursen et al. 1980). Polyploids of *Hordeum brevisubulatum* are thought to be autoploid based on morphological (Bothmer 1979, Dewey 1979) and hybrid meiotic analysis (Landström et al. 1984).

In this paper, the molecular karyotype of the I genome is described in detail based on physical mapping of all possible dinucleotide and trinucleotide SSRs along with 5S rDNA, 45S rDNA, pSc119.2, and pAs1 repeats on mitotic chromosomes in *Hordeum bogdanii* and *Hordeum brevisubulatum*. The results provide more information on the genomic differentiation in *Hordeum* at the chromosomal level and will also help elucidate the functional and evolutionary implications of different repetitive sequences as genomes differentiate during speciation.

## Material and methods

### Plant material

*Hordeum bogdanii* was collected in Germu, Qinghai, China. Hordeum brevisubulatum ssp. brevisubulatum was collected in Tongde, Qinghai, China. More than 50 or more than 100 individuals of both species were collected in the field. Samples used for cytogenetic investigation were randomly selected from different individuals. Approximately 20 individuals were used for chromosome preparation. Only the investigated samples that displayed clear FISH patterns were present in this study.

### Slide preparation

Seeds of *Hordeum bogdanii* and *Hordeum brevisubulatum* were germinated on moist filter paper in petri dishes at room temperature. Root tips with a length of approximately 1–2 cm were excised, pretreated in N_2_O gas for 2 h as described by Kato (1999), and fixed in 3:1 (v/v) 100% ethanol:glacial acetic acid. Each root tip was squashed in a drop of 45% acetic acid.

### DNA probes and labelling

Synthetic dinucleotide SSRs (AT)_15_, (AG)_15_, (AC)_15_, and (GC)_15_ and trinucleotide SSRs (AAG)_10_, (AAC)_10_, (AAT)_10_, (ACG)_10_, (ACT)_10_, (AGG)_10_, (CAC)_10_, (CAG)_10_, (CAT)_10_ and (GGC)_10_ were end-labelled with fluorescein amidate (FAM, green, Sangon Biotech Co., Ltd., Shanghai, China). For the repetitive sequences pAs1 (Rayburn and Gill 1986), pSc119.2 (Bedbrook et al. 1980), and 45S rDNA, oligonucleotide probes described by Danilova et al. (2012) and Tang et al. (2014) were used. The oligonucleotides pSc119.2 and 45S rDNA were end-labelled with FAM (green), and pAs1 was end-labelled with TAMRA (red). The 5S rDNA was amplified by polymerase chain reaction (PCR) using genomic DNA of *Hordeum bogdanii* as described by Fukui et al. (1994) and was labelled with fluorescein-12-dUTP (green) using a random primer labelling method. Genomic DNA of *Hordeum bogdanii* was labelled with tetramethy1-rhodamine-5-dUTP using the random primer method, as described by Dou et al. (2009).

### FISH and microphotometry

FISH experiments were conducted using the method of Dou et al. (Dou et al. 2009) with minor modifications. Samples on prepared slides were denatured in 0.2 M NaOH in 70% ethanol at room temperature for 10 min, rinsed in 70% cold ethanol (stored at minus 20°C) for approximately 30 minutes, and air dried. The hybridization mixture, 10 µl per slide, was prepared by adding 50% de-ionized formamide, 50% dextran sulphate, 2 × SSC (0.3 M NaCl, 0.03 M Na3-citrate), 1 µg/µl denatured salmon sperm DNA and 10 ng of probe. The hybridization mixture with oligonucleotide probes was placed directly onto the denatured slide preparation. The hybridization mixture with the 5S rDNA probe was denatured in boiling water for 5 minutes. Hybridization was conducted overnight at 37°C in a moistened chamber. Chromosomes were counterstained with 4',6-diamidino-2-phenylindole (DAPI). Images were captured with a cooled CCD camera (Photometrics CoolSNAP) using a fluorescence microscope (Leica) and were processed with the Meta Imaging System (Universal Imaging Corporation). Finally, images were adjusted using Adobe Photoshop 6.0 for contrast and background optimization.

## Results

### Chromosomal organization of repeats in *Hordeum bogdanii*

**Figure 1.**
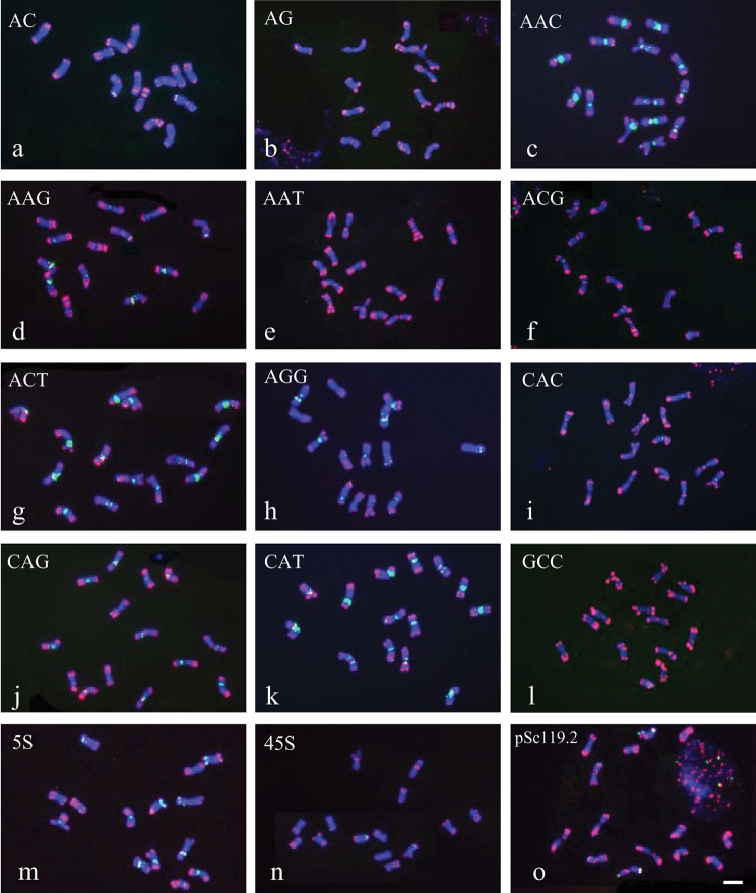
FISH patterns of mitotic metaphase chromosomes of *Hordeum bogdanii* detected by pAs1 (red) combined with the several other repeats (green): **a** (AC)_15_ **b** (AG)_15_ **c** (AAC)_10_ **d** (AAG)_10_ **e** (AAT)_10_ **f** (ACG)_10_ **g** (ACT)_10_ **h** (AGG)_10_ **i** (CAC)_10_ **j** (CAG)_10_ **k** CAT)_10_ **l** (GCC)_10_ **m** 5S rDNA **n** 45SrDNA **o** pSc119.2. Bar = 10 µm.

**Figure 2.**
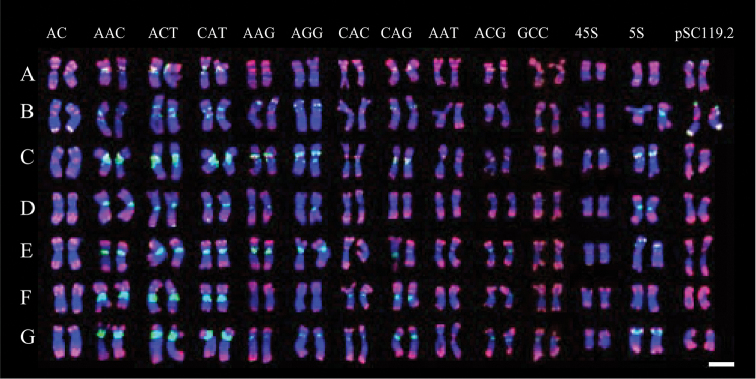
Molecular karyotypes of *Hordeum bogdanii* probed by pAs1 (red) combined with the other several repeats (green). Seven pairs of chromosomes are designated from **A–G** for distinguishing them from the numerals of the 7 homologue groups used in barly. Bar =10 µm.

A stable chromosome number of 2n = 14 was detected in all tested samples of *Hordeum bogdanii*. The repetitive sequence pAs1 produced multiple sites that were subtelomeric, intercalary, or pericentromeric on all chromosomes. The hybridization pattern of pAs1 was informative enough to distinguish each chromosome of *Hordeum bogdanii*. Thus, chromosome localization of other repeats was conducted using pAs1 as a landmark (Fig. 1). The chromosomal distribution of each sequence was described in detail (Fig. 2).

The four possible dinucleotide SSR probes (AG)_15_, (AC)_15_, (AT)_15_, and (GC)_15_ were used to characterize the chromosomes of *Hordeum bogdanii*. Only (AC)_15_ produced detectable hybridization signals, which appeared in subtelomeric regions on all chromosomes and in pericentromeric regions on a few chromosomes. The signals were strongest in subtelomeric regions of chromosomes B, C and D (Fig. 2).

All 10 trinucleotide SSRs produced detectable hybridizations (Fig. 2). The probe (AAC)_10_ revealed many intense and rich pericentromeric hybridization signals on all seven pairs of chromosomes, intercalary signals on chromosomes B and F, and subtelomeric signals on chromosomes D and E. The FISH patterns of (ACT)_10_ and (CAT)_10_ appeared to be identical to those of (AAC)_10_. An identical FISH pattern was shared by (AAG)_10_ and (AGG)_10_, which produced intense pericentromeric hybridizations, intercalary hybridizations, or both in six pairs of chromosomes. The (CAC)_10_ probe produced faint signals in four chromosome pairs and strong signals in three pairs in pericentromeric regions, intercalary regions, or both. The distribution of the (CAG)_10_ signal overlapped with that of the (CAC)_10_ signal, but the (CAG)_10_ signal had a stronger hybridization intensity. (AAT)_10_ produced faint signals in three pairs of chromosomes in pericentromeric, intercalary, and subtelomeric regions. Hybridization of (ACG)_10_ was detected in six pairs of chromosomes and was localized primarily in centromeric regions. The distribution of (GCC)_10_ was more dispersed; however, more intense hybridizations were still observed in centromeric regions on three pairs of chromosomes.

Three 45S rDNA sites were detected in two pairs of chromosomes. One carried a distinct site on the subtelomeric region of the short arm; the other harboured two faint hybridization sites in the ends of both arms. 5S rDNA was distributed in all chromosomes except for one. Nine distinct 5S rDNA sites were exclusively determined and were localized on centromeric, pericentromeric, intercalary, or subtelomeric regions. Three pairs of chromosomes carried only one 5S rDNA site, and another three harboured two 5S rDNA sites. Two 45S rDNA and three 5S rDNA sites were reported in an accession of *Hordeum bogdanii* (Taketa et al. 2001). The fact that many more 45 S rDNA and 5S rDNA sites were revealed in this study suggests the presence of intra-specific polymorphisms. Four distinct hybridization sites were probed by pSc119.2, and these were localized on subtelomeric regions of three pairs of chromosomes.

### Chromosomal organization of repeats in *Hordeum brevisubulatum*

**Figure 3.**
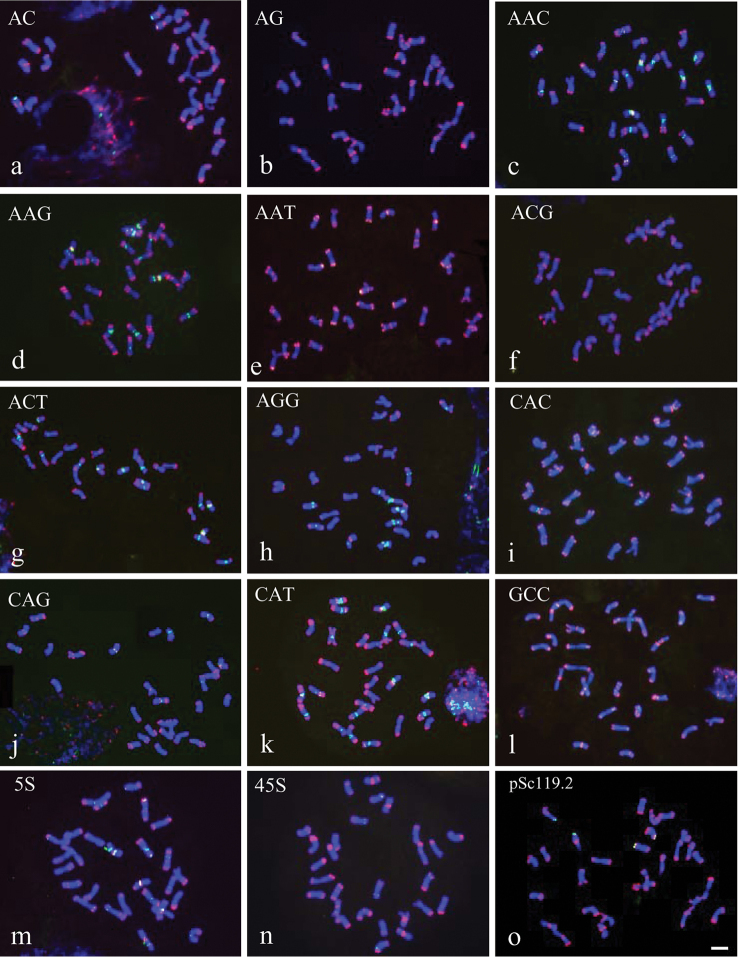
FISH patterns of mitotic metaphase chromosomes of *Hordeum brevisubulatum* detected by pAs1 (red) combined with several other repeats (green): **a** (AC)_15_ **b** (AG)_15_ **c** (AAC)_10_ **d** (AAG)_10_ **e** (AAT)_10_ **f** (ACG)_10_ **g** (ACT)_10_ **h** (AGG)_10_ **i** (CAC)_10_ **j** (CAG)_10_ **k** (CAT)_10_ **l** (GCC)_10_ **m** 5S rDNA **n** 45SrDNA **o** pSc119.2. Bar = 10 µm.

**Figure 4.**
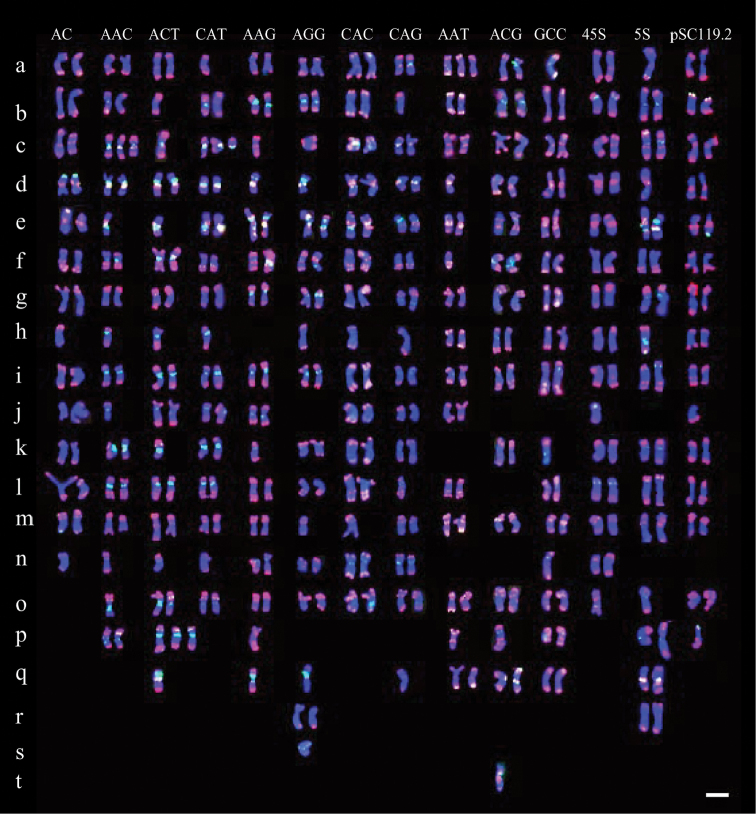
Molecular karyotypes of *Hordeum brevisubulatum* probed by pAs1 (red) combined with several other repeats (green). Chromosome types are designated by lowercase roman letters from a to t to distinguish them from the symbols designating *Hordeum bogdanii*. Bar = 10 µm.

A chromosome number of 2n = 28 was detected in nearly all tested individuals of *Hordeum brevisubulatum*. However, chromosome numbers of 2n = 26 and 2n = 27 (Fig. 3a, d) were also observed in a few cases. Moreover, a monotelosomic chromosome was clearly identified in one case (Fig. 3k). Karyotyping was tentatively conducted using pAs1 combined with other repeats. Highly variable karyotypes were revealed in *Hordeum brevisubulatum* (Fig. 4). Variations in chromosome number, such as monosomy, nullisomy, and trisomy, were clearly shown to have occurred. Chromosome structural aberrations, such as deletions, translocations, or inversions, are likely to be present, although these aberrations were not evident from the FISH results alone. Thus, approximately 20 chromosomal variants designated from a to t were roughly identified in the individuals that were investigated (Fig. 4).

All possible di- and trinucleotide SSR probes except for (AG)_15_, (AT)_15_ and (GC)_15_ produced hybridizations on chromosomes of *Hordeum brevisubulatum*. The (AC)_15_ probe was primarily detected in subtelomeric regions. Nearly half of the total chromosomes carried detectable signals, and signals of seven or eight chromosomes appeared more distinct. The chromosomal distribution of (AAC)_10_, (ACT)_10_, and (CAT)_10_ was similar to that in *Hordeum bogdanii*. The (AAC)_10_, (ACT)_10_, and (CAT)_10_ repeats were still colocalized and were detected on 22–26 chromosomes. (AAG)_10_ and (AGG)_10_ appeared to be colocalized and were primarily distributed in pericentromeric regions on 13–16 chromosomes. (CAC)_10_ and (CAG)_10_ had similar distributions to those observed in *Hordeum bogdanii*. Signals of (CAC)_10_ and (CAG)_10_ were detectable in 19–22 chromosomes and were stronger on eight or nine chromosomes. The FISH pattern of (AAT)_10_ in *Hordeum brevisubulatum* was distinctly different from that in *Hordeum bogdanii*. (AAT)_10_ produced a stronger hybridization signal in *Hordeum brevisubulatum* than in *Hordeum bogdanii*, primarily in subtelomeric regions on nearly all chromosomes. Signals of (ACG)_10_ were detectable in 13 chromosomes. As with (AAT)_10_, stronger hybridization signals of (GCC)_10_ were detected in *Hordeum brevisubulatum* than in *Hordeum bogdanii*.

45S rDNA produced hybridization signals in subtelomeric regions on seven or eight chromosomes. 5S rDNA was detected in centromeric, pericentromeric, intercalary, or subtelomeric regions on 24 chromosomes. The present karyotypes of 5S rDNA and 45S rDNA in *Hordeum brevisubulatum* differed from those of *Hordeum brevisubulatum* based on the accessions reported by Taketa et al. (1999). A heterogeneous composition of 5S and 45S rDNA was observed in a total of five 5S rDNA sites and 18 45S rDNA sites (Taketa et al. 1999). However, many more 5S rDNA sites than 45 S rDNA sites were detected in our study. This pattern suggests that intraspecific polymorphism may exist among different populations. Distinct pSc119.2 signals were detected in subtelomeric regions on four or five chromosomes.

**Figure 5.**
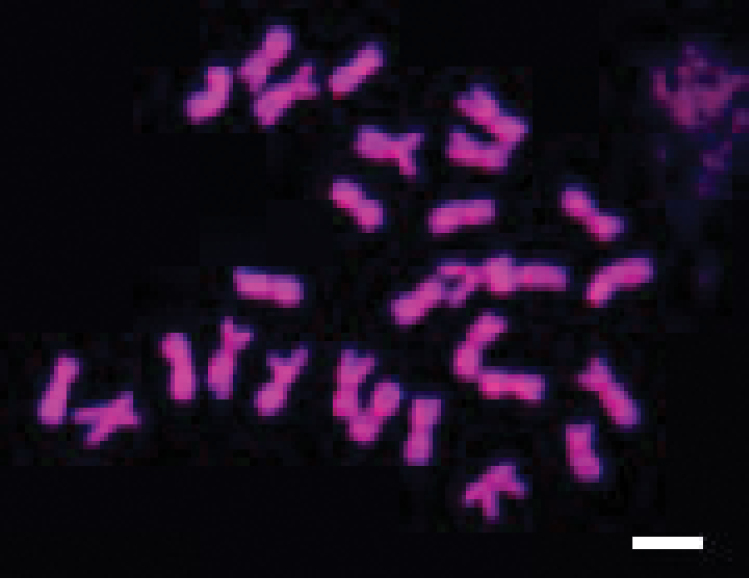
GISH patterns of mitotic metaphase chromosomes of *Hordeum brevisubulatum* probed by the genomic DNA of *Hordeum bogdanii*.

Furthermore, the technique of genomic *in situ* hybridization (GISH) was used to determine the polyploidy origin of *Hordeum brevisubulatum*. The result showed that the chromosomes of *Hordeum brevisubulatum* were evenly painted by using labelled genomic DNA of *Hordeum bogdanii* as the probe (Fig. 5).

## Discussion

### Chromosomal identification and genomic characterization of *Hordeum bogdanii*

**Figure 6.**
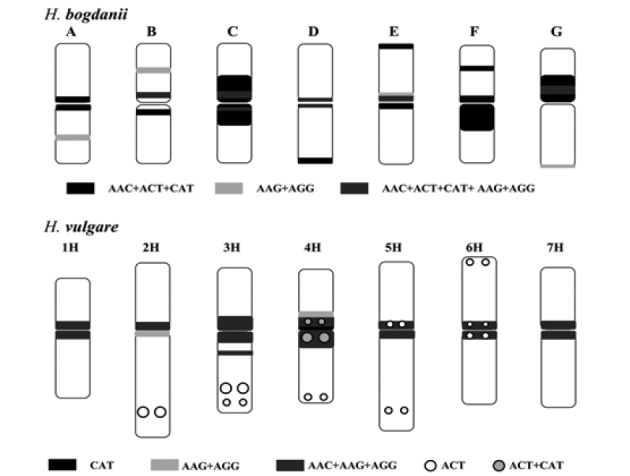
Idiogram of chromosomes of *Hordeum bogdanii* (I genome) and *Hordeum vulgare* (H genome) showing the distributions of the SSRs AAC, ACT, CAT, AAG and AGG. Chromosomal information of *Hordeum vulgare* is taken from Cuadrado et al. (2007a)

Fifteen of the 18 repetitive sequences produced detectable hybridization in mitotic metaphase chromosomes in *Hordeum bogdanii*. A few repeats, including highly polymorphic sites, can be used to uniquely identify each chromosome. The satellite DNA pAs1 and 5S rDNA can be used to distinguish each chromosome in *Hordeum bogdanii*. The SSRs AAC, ACT, CAT, AAG and AGG are also ideal markers for chromosome identification because of their abundance and their large number of polymorphic sites across individual chromosomes (Fig. 6). Thus, individual chromosomes of *Hordeum bogdanii* can be easily identified using the above repeats. In this study, pAs1 repeats were used as a reference marker, and each repetitive sequence was accurately physically mapped.

Most of the repeats produced multiple hybridizations. However, high-intensity hybridizations were always observed on pericentromeric or subtelomeric regions. This implies that the *Hordeum bogdanii* genome contains more repetitive sequences in subtelomeric and pericentromeric parts of the chromosome than in interstitial regions. The distributions of AAC, ACT, and CAT were revealed to be colocalized and were identical in intensity in this study, suggesting that AAC, ACT, and CAT may be evenly distributed in an intermingled way. The same was true for AAG and AGG. Although CAC and CAG were found to be colocalized, different hybridization intensities suggest that their distribution was close rather than intermingled.

Differences between the I and H genomes can be observed by comparing their distribution of their repetitive sequences. A distinct difference can be observed in a few trinucleotide SSR motifs. In *Hordeum vulgare* (H genome), the AAC, AAG, and AGG motifs are colocalized around the centromere in all chromosomes; ACT produces multiple signals in six chromosome pairs; and CAT produces a strong signal and a weak signal in 4H and 5H, respectively (Cuadrado and Jouve 2007a) (Fig. 6). Another distinct difference is exhibited by the dinucleotide AG, which was intensely detected around the centromere in *Hordeum vulgare* (Hagras et al. 2005, Carmona et al. 2013a) but was absent in *Hordeum bogdanii*. Additional differences between *Hordeum bogdanii* and *Hordeum vulgare* can be seen in the distribution patterns of AAT, GCC, CAG, 45S rDNA, 5S rDNA and pSc119.2. Physical mapping of a few sequences in *Hordeum marinum* (Xa genome) showed that AAC and AAG produced intense and rich patterns of multiple SSR signals that were particularly concentrated in the pericentromeric region and that ACT and CAT were weakly distributed (Carmona et al. 2013b). The pSc119.2 repeat produced sub-telomeric signals on nearly all chromosomes in *Hordeum marinum* (Taketa et al. 2000); however, fewer of these signals were present in *Hordeum bogdanii*. Karyotype analysis of diploid *Hordeum murinum* (Xu) showed that AG produced intense signals around the centromere in four chromosome pairs and that AAG produced more rich and intense signals than AAC, ACT, and CAT in pericentromeric regions (Cuadrado et al. 2013, Carmona et al. 2013b). In addition, no pSc119.2 hybridization signals were detected in diploid *Hordeum murinum* (Taketa et al. 2000).

Thus, the genomic composition of the I genome in *Hordeum bogdanii* revealed by the distribution of several repeats was highly different from that of the H, Xa, and Xu genomes in *Hordeum*.

### Autopolyploid origin of *Hordeum brevisubulatum*

The *Hordeum brevisubulatum* species complex has a range of cytotypes and can occur in diploid, tetraploid, and hexaploid forms (Bothmer 1979, Dewey 1979, Linde-Laursen et al. 1980). Earlier studies have shown that polyploids in the *Hordeum brevisubulatum* complex may be autoploids (Bothmer 1979, Dewey 1979). Data from meiotic pairing in the hybrids indicate that autoploidy characterizes the entire complex, with one “basic” genome (Landström et al. 1984). The karyotype of tetraploid *Hordeum brevisubulatum* was not shown to be a strictly doubled karyotype from a diploid form. However, similar chromosomal distributions of many repetitive sequences between *Hordeum bogdanii* and *Hordeum brevisubulatum* were revealed in this study. Specifically, colocalized distributions of AAC, ACT, and CAT in pericentromeric regions in nearly all chromosomes and colocalized distributions of AAG and AGG in most chromosomes were observed in *Hordeum brevisubulatum*. Nearly double the number of 45S rDNA and 5S rDNA sites in *Hordeum bogdanii* were also detected in *Hordeum brevisubulatum*. In addition, no genomic differentiation was detected in *Hordeum brevisubulatum* by GISH with labelled genomic DNA of *Hordeum bogdanii*. This pattern strongly suggests an autopolyploid origin of *Hordeum brevisubulatum* from an I genome.

Several examples of structural rearrangements of chromosomes at the population level in the *Hordeum brevisubulatum* complex have been reported (Landström et al. 1984). In this study, high levels of chromosomal variation, including variation in chromosome number and chromosome structure, were observed in the population under investigation. This observation implies that there is a high degree of instability of the autoploid genome in *Hordeum brevisubulatum*. Karyotype variations have recently been reported in resynthesized and naturally formed allopolyploid species and in natural hybrids produced by polyploid homoploid hybridization (Chester et al. 2012, Lipman et al. 2013). Aneuploidy, inter- and intragenomic rearrangements, and the loss of repeats were frequently detected in early generations. As a natural species, *Hordeum brevisubulatum* should be the product of a long evolutionary history. *Hordeum brevisubulatum* has been considered to be self-incompatible (Bothmer 1979). Although autopolyploidy may result from genome doubling within a single individual, most natural autopolyploids likely formed via some degree of hybridization, involving, for example, individuals from genetically differentiated populations (Stebbins 1985, Soltis and Soltis 1989). Thus, more studies are needed to determine whether karyotype variation in *Hordeum brevisubulatum* might be affected by hybridization between genetically differentiated biotypes.

Major genetic changes, including the loss of homologs and DNA sequences, have been documented in recently formed polyploid species (Soltis et al. 2004, Abbott and Lowe 2004, Matyášek et al. 2007). Compared with the composition of the I genome in *Hordeum bogdanii*, the distributions of a few repetitive sequences in *Hordeum brevisubulatum* might be strongly altered by increases or decreases in copy number. The trinucleotide SSR motifs AAC, ACT, and CAT are primarily located in pericentromeric regions in all the chromosomes in *Hordeum bogdanii*, but they are absent in a few chromosomes in *Hordeum brevisubulatum*, possibly because of decreases in copy number. The distributions of AAG and AGG are similar. In contrast, the higher abundance of ATT and GCC in *Hordeum brevisulatum* indicates that there is an increase in copy number that accompanies polyploidization.

### Evolutionary trends of repetitive DNA sequences during genome differentiation

Repetitive DNA sequences are the main components of heterochromatin and are subject to rapid change. Such changes in the distribution of repetitive DNA sequences are one of the driving forces of genome evolution and speciation. One proposed function of repetitive sequences may be related to higher-order molecular structure (Redi et al. 2001). However, the molecular mechanisms by which genomes change are unknown (Plohl et al. 2002).

The SSR motif AAC was shown to be distributed mainly in the pericentromeric regions of the I, H, Xa, and Xu genomes. In addition, the wide distribution of AAC has been detected in wheat and the genus *Secale* (Cuadrado et al. 2000 Cuadrado and Jouve 2002). This wide distribution suggests that AAC repeats have an ancient origin in Triticeae species. The distribution of AAC in the I genome was revealed to be evenly co-localized with ACT and CAT. However, the distributions of AAC, ACT, and CAT are largely differentiated in the H, Xa, and Xu genomes. Variation in the distribution of repetitive sequences may be related to the amplification and deletion of repeat copies. AAC, ACT, and CAT motifs in the I genome may have been co-amplified or deleted as a single repetitive unit. However, AAC, ACT, and CAT in other genomes have evolved independently. AAG exhibits the most chromosomal variation both within and between taxa (Pedersen et al. 1996, Pedersen and Langridge 1997, Cuadrado et al. 2008, Carmona et al. 2013a). AAG has been suggested to be more predisposed to being amplified or deleted relative to other repetitive sequences as a consequence of independent events in different lineages (Carmona et al. 2013b). The fact that AAG is evenly colocalized with AGG in the I genome suggests that the same evolutionary mechanism also drives variation in the chromosomal distribution of AAC, ACT, and CAT.

AC repeat sites are remarkably similar and have been shown to exhibit uniformly dispersed hybridization along the euchromatic portion of metaphase chromosomes in humans and barley and in the metaphase and polytene chromosomes of *Drosophila melanogaster* (Meigen, 1830) (Cuadrado and Jouve 2007b). In this study, AC repeat sites had a dispersed distribution. High-intensity hybridization bands for AC repeats were observed in subtelomeric and pericentromeric regions on a few chromosomes in the I genome. This atypical distribution suggests that there is a more complicated organization or function of AC repeats in plant genomes.

The sequence pAs1 contains Afa family sequences. The Afa family sequence was abundant in the four basic genomes of the genus *Hordeum*, and the hybridization patterns differed among the diploid species (Taketa et al. 2000). The distribution of the Afa family is more dispersed than those of the other tandem repetitive sequences. This dispersed distribution implies that the Afa family may be more commonly distributed in gene rich regions. In our study, despite the autopolyploidy origin of *Hordeum brevisubulatum* from an I genome species, the distribution of pAs1 sequences in *Hordeum brevisubulatum* was highly variable relative to those in diploid *Hordeum bogdanii*. The Afa family of sequences may play an important role in the differentiation of either genome or species in the genus *Hordeum*.

Variation in the abundance and distribution of repetitive sequences and the shared distribution of the same repeats between different genomes suggest that repetitive sequences play a key role in both the structure and function of the genomes of higher eukaryotes (Cuadrado et al. 2008). To date, most information on the distribution of repeats in *Hordeum* genomes comes from physical mapping analysis of mitotic metaphase chromosomes, which are highly packaged. Physical mapping of repetitive sequences on extended chromosomes, such as during the pachytene stage of meiosis, may provide more valuable information on the chromosomal distribution of repeats and may help elucidate the evolution and genomic function of repetitive sequences.

## Conclusion

Fifteen repetitive sequences, including SSR motif AC and all possible trinucleotide motifs and satellite DNAs pAs1, 5S rDNA, 45s rDNA, and pSc119.2, were accurately physically mapped on individual chromosomes in the I genome in *Hordeum bogdanii*. High genome instability was revealed in tetraploid *Hordeum brevisubulatum*. The similar distribution of the repeats in both species suggests an autopolyploid origin of *Hordeum brevisubulatum* from an I genome species. Comparative cytogenetic analysis between the I genome and other genomes in *Hordeum* showed that the distribution of a few repeats differed. Colocalization of motifs AAC, ACT, and CAT and colocalization of motifs AAG and AGG is characteristic of the I genome.
